# Challenges of proper disposal of old long-lasting insecticidal nets and its alternative uses in rural south-eastern Tanzania

**DOI:** 10.1371/journal.pone.0279143

**Published:** 2024-02-15

**Authors:** Sheila J. Msangi, Winifrida P. Mponzi, Letus L. Muyaga, Joel D. Nkya, Yohana A. Mwalugelo, Hajirani M. Msuya, Dickson W. Lwetoijera, Emmanuel W. Kaindoa

**Affiliations:** 1 Environmental Health and Ecological Sciences Department, Ifakara Health Institute, Ifakara, Tanzania; 2 School of Life Sciences and Bio Engineering, The Nelson Mandela African Institution of Science and Technology, Arusha, Tanzania; 3 Institute of Biodiversity, Animal Health and Comparative Medicine, University of Glasgow, Glasgow, United Kingdom; 4 Department of Biomedical Sciences, Jaramogi Oginga Odinga University of Science and Technology, Bondo, Kenya; 5 Faculty of Health Sciences, School of Pathology, National Institute for Communicable Diseases, University of the Witwatersrand and the Centre for Emerging Zoonotic and Parasitic Diseases, Johannesburg, South Africa; University of Uyo, NIGERIA

## Abstract

**Introduction:**

Insecticide-treated nets (ITNs), specifically long-lasting insecticidal nets (LLINs), are the most commonly used, scalable, and cost-effective tools for controlling malaria transmission in sub-Saharan Africa. However, the multiple alternative uses of retired LLINs have been associated with poor disposal practices. The World Health Organization (WHO) has provided guidelines and recommendations for the proper management of worn-out LLINs. This study assessed the existing alternative uses and disposal practices of old LLINs.

**Methods:**

An explanatory sequential mixed-methods approach was used to assess LLINs existing alternative uses, disposal practices, knowledge, and perceptions regarding WHO recommendations on proper disposal of old LLINs among stakeholders in Kilombero and Ulanga districts, south-eastern Tanzania. A survey questionnaire was administered to 384 participants. Furthermore, the study employed focus group discussions (FGD) and key informant interviews (KII) to elucidate responses regarding existing disposal practices, associated challenges, and alternative uses of LLINs. The insights derived from both study components were subsequently used for inferential analysis.

**Results:**

The major challenge influencing the proper disposal of LLINs was limited awareness of how to properly dispose of them. Of the 384 people surveyed, 97.0% were not aware of the WHO recommendations for the proper disposal of old LLINs. All key informants were unaware of the WHO guidelines for proper disposal of old LLINs. The common methods used to dispose of LLINs were burning (30.7%), disposing them into garbage pits (14.8%), and alternative uses (12.2%). Of the 239 respondents with LLINs, 41.0% had alternative use, while 59.0% had no alternative use. The common alternative uses were ropes for tying or covering items (20.9%), garden fencing (7.5%), chicken coops (5.0%), and 7.5% for other minor alternative uses.

**Conclusion:**

Strengthening awareness and education on proper LLIN disposal practices among community members and key stakeholders is essential for enhancing malaria control efforts and preventing environmental pollution.

## Introduction

Globally, more than 2.3 billion insecticide-treated nets (ITNs) were distributed between 2004 and 2020, and sub-Saharan Africa (SSA) received two billion (86%) of all distributed insecticide-treated nets [1]. Tanzania is among the top five recipients [2]. This massive volume of ITNs, particularly long-lasting insecticidal nets (LLINs), placed in circulation could lead to a rapid increase in solid waste litter and environmental pollution in SSA countries, where solid waste management remains a major challenge [3–5]. LLINs are among the possible sources of plastic waste, but their ultimate disposal and environmental impacts are yet to be determined. Hence, the growing utilization of LLINs may present a dilemma regarding the proper disposal of retired bed nets. The primary aim of using LLIN is to protect people against mosquito bites, malaria transmission, and other mosquito-borne infections [6]. However, in several parts of Africa, LLINs have been used for fencing vegetable gardens and chicken huts, sieving grains, fishing nets, and alternative building materials, which may cause environmental harm [7–11]. The insecticides (pyrethroids and pyrroles) embedded in LLINs exhibit low toxicity to mammals; however, improper disposal could pose a risk to aquatic ecosystems and non-targeted species. These insecticides may exert additional insecticide selection pressure on mosquitoes in their aquatic habitats [12]. Thus, any form of repurposing should consider protecting such life as stipulated in the Sustainable Development Goals (SDGs) 14 and 15 on life below water and life on land, respectively [13].

Plastic pollution is a pressing public health concern because of its prevalence, long-lasting nature, persistence in the environment, and perceived risks to living organisms [14]. Environmental pollution resulting from plastic materials continues to be a global challenge due to threats to aquatic life, wildlife, climate change, human health, and economic development [15–19]. Despite the adoption of an anti-plastic bag policy in various countries in SSA [4], there have been insufficient measures against LLINs and their packages and bag disposal. The World Health Organization (WHO) has provided warnings on the possible impact of accumulated LLINs and their packages. This may include contamination of crops, vegetables, soil, and groundwater when these LLINs are reused, consequently producing dangerous persistent toxins resulting from open-air burning [20]. Thus, the development of environmentally friendly programs for proper disposal of LLINs and their packing bags is critical and should be explored to ensure adherence to the current legislation specified by environmental management authorities. The WHO has recommended proper ways of disposing or handling old LLINs, namely: 1) continuing to be used even if they have holes until they are replaced with new LLINs; 2) old LLINs should not be disposed of in water bodies; 3) collection of the old LLINs for disposal by NMCP; 4) incineration of old LLINs; and 5) formulating guidelines, policies, and regulations by the NMCP in collaboration with national environmental authorities [20, 21]. An important question is whether local health and environmental authorities are aware of such guidelines and whether they have the capacity to implement and monitor them. Nevertheless, information from various sources consistently highlights high levels of ITN attrition from households [22], indicating high concerns about the accumulation of old nets in the environment.

The issue of high attrition rates for LLINs, which are intended to last for three years but often deteriorate prematurely, coupled with the problem of oversupply due to frequent net replacements, underscore the urgent need for an improved intersectional approach. To address this challenge, stakeholders, including international organizations, governments, NGOs, and communities, must work collaboratively to enhance education on net care and maintenance, ensure the distribution of high-quality nets, and coordinate resource allocation to areas with the greatest malaria burden.

The inaccessibility of clear information from international agencies due to communication gaps or limited readily available information on this topic poses significant challenges for researchers and practitioners seeking authoritative guidance on the proper disposal of Long-Lasting Insecticidal Nets (LLINs). However, while information from relevant authorities, including the WHO, the United Nations Environment Programme (UNEP), and the Basel, Rotterdam, and Stockholm Conventions on the sound management of old LLINs is available, there is evidence indicating that old LLINs are often misused and improperly disposed of within communities. This misuse includes repurposing LLINs for activities other than their intended use [9]. Thus, this study was conducted to assess the existing alternative uses and disposal practices of LLINs as well as community members and key stakeholders’ knowledge and perceptions regarding the WHO recommendations on proper disposal of LLINs in selected communities in Tanzania. Therefore, understanding the factors influencing poor disposal practices and associated alternative uses would be beneficial for decision making and policymakers.

## Methods

### Study area

**Fig 1:**
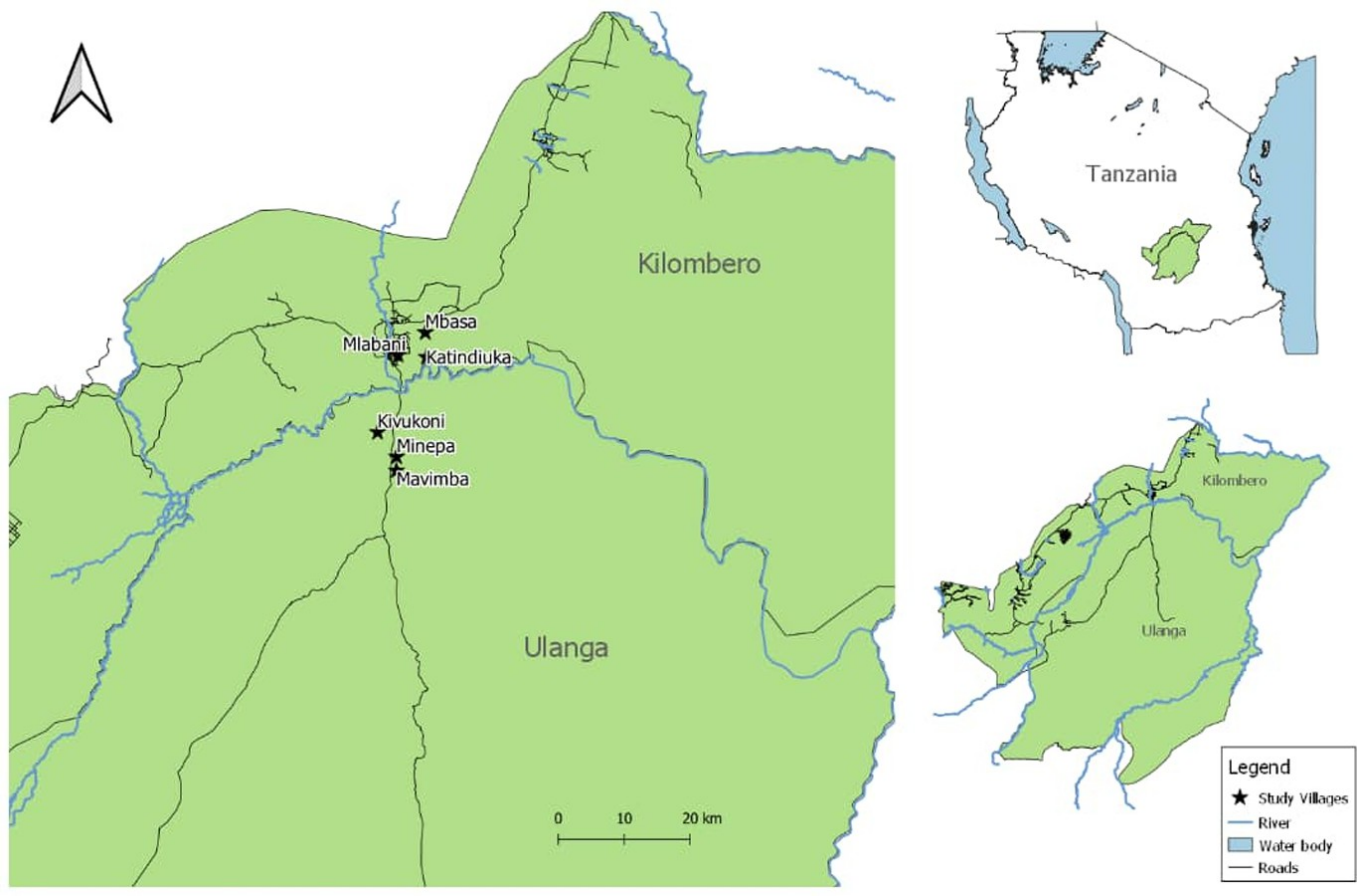
A map of Kilombero and Ulanga districts in south-eastern Tanzania showing study wards.

This study was conducted in six wards in Kilombero and Ulanga districts, both in the Kilombero valley in south-eastern Tanzania (Fig 1). In Ulanga district, the study was done in Kivukoni, Mavimba, and Minepa villages. In semi-urban settings of the Kilombero district, the study was done in Ifakara town council and its surrounding wards, including Katindiuka, Mlabani, and Mbasa. The economic activities in the study sites are predominantly rice farming, retail businesses, and fishing. The major mosquito control intervention is use the of LLINs that are freely distributed at antenatal clinics and the school net program (SNP) and universally distributed by the government every 3–4 years [23].

### Study design and data collection procedure

**Fig 2:**
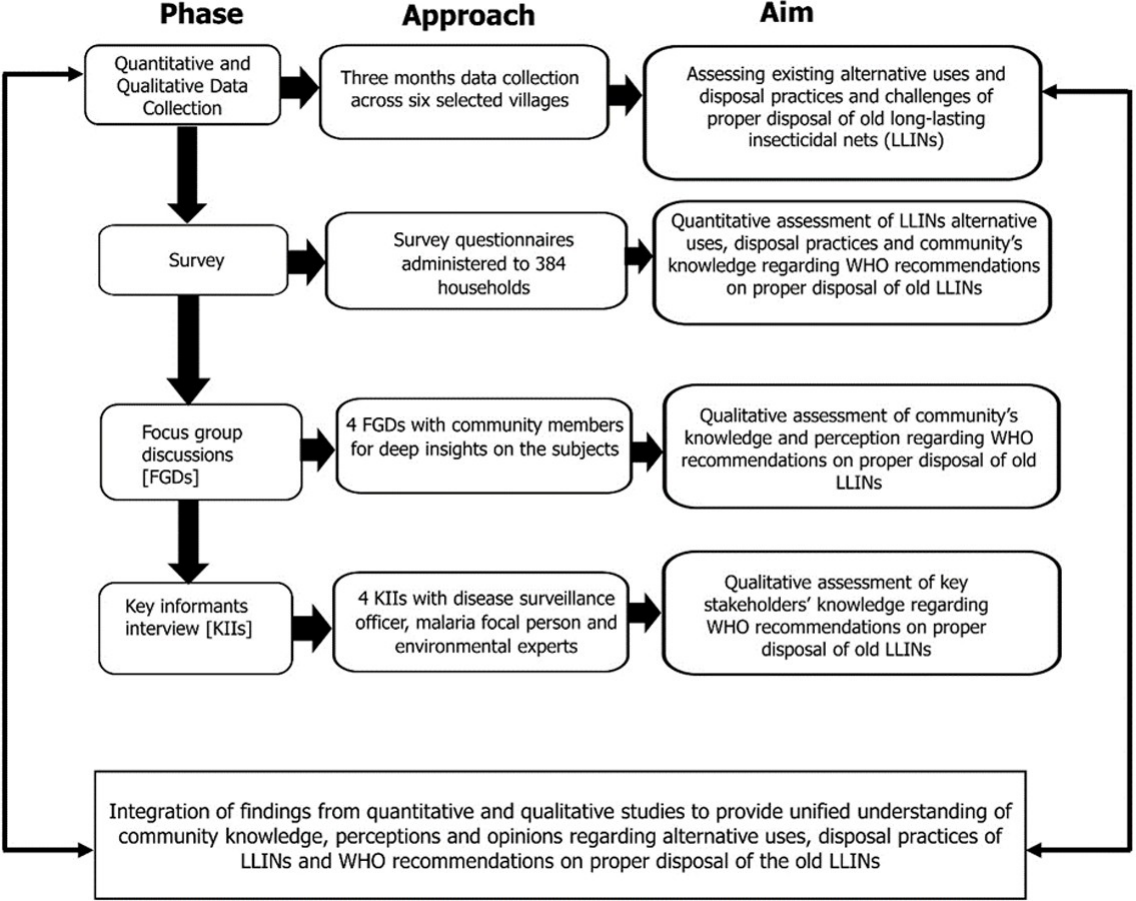
Illustration of the explanatory sequential mixed methods approach used to examine community knowledge and perceptions of alternative uses, disposal practices and challenges and WHO recommendations regarding proper disposal of old LLINs.

This study was an explanatory sequential mixed-method design [24, 25] where quantitative data analysis provided insights for qualitative study themes. This approach had two components: the first was a quantitative survey of 384 households in two districts. The second component consisted of four focus group discussions (FGDs) with community members selected from survey respondents (two groups with males and two with females), followed by four key informants from the public health and environmental sectors. The qualitative findings were used to clarify some of the responses from the initial survey questionnaire (Fig 2). The findings from the two components were used to make inferences.

### Survey questionnaire

The sample size for the survey questionnaire was determined using the Epi-Info Software package [26–28] based on the following assumptions: sample size based on the single proportion for cross-sectional survey assumed; 50% of the population would dispose of or/and alternative uses of bed nets, with 5% margin of error at 95% confidence level. Thus, a sample size of 384 was reached. The households were randomly selected from household list available at the ward offices and community leaders from respective wards. To ensure a balanced design, 64 households were randomly selected in each ward (making up 384 households for the six selected study areas) and consented to participate in the survey.


$$\begin{array}{l} N=\frac{1.96^{2}*(0.5)(1-0.5)}{0.05^{2}}\\ N=384 \end{array}$$

A survey questionnaire was translated into Swahili and administered using electronic forms on a free-access application programmed on Open Data Kit (ODK) from the Kobo ToolBox server [29]. Later, the survey was translated back from Swahili into English. To ensure accuracy and effectiveness, the questionnaire was pilot-tested on ten potential participants, and necessary revisions were made based on their feedback.

The study participants’ inclusion criteria for the survey questionnaire and focus group discussions (FGDs) were set to include male and female household heads or representatives aged 18–80 years of sound mind who; (1) had a LLIN being used for alternative uses in their compound or garden, and (2) whose compound or garden had no alternative uses practices.

### Focus group discussions

Four (4) focus group discussions (FGDs) were held in May 2022 with a subset of survey respondents to clarify the findings derived from the quantitative component. Participants were purposefully selected; they included those with or without LLINs and those who had alternatively used them or not. The discussions consisted of two male groups and two female groups in four wards; each session had 7–8 participants. To maximize participation, male and female participants were separated during the discussions. The discussions provided detailed information on community members’ understanding and perceptions of alternative uses of LLINs, their disposal practices, and awareness and understanding of WHO guidelines on proper management of old LLINs. The topics explored in the FGDs included the experiences and perceptions of the participants on 1) LLIN use, care, and repair; 2) LLIN damage and changing decisions; 3) LLIN disposal and alternative uses; and 4) WHO guideline awareness on the sound management of old LLINs. The FGD guide was pre-tested, and questions were refined according to the outcomes. The guide was then translated from Swahili into English. The discussions were held at local ward offices or in classrooms at local primary schools. The discussions were audio-recorded, and specific notes were taken. The discussions were held in Swahili, Tanzania’s native language.

### Key informant interviews

Key informant interviews (KIIs) were conducted with four purposively selected stakeholders from the public health and environmental sectors due to their direct or indirect involvement in malaria, vector control, and environmental management. The KIIs included the Ifakara disease surveillance officer, the Ulanga malaria focal person, the Ifakara environmental officer, and the Ulanga environmental health officer. These informants were interviewed to investigate their knowledge regarding WHO recommendations as well as their awareness of ongoing bed net alternative uses and disposal practices. The discussions provided in-depth information on key stakeholders’ perceptions and perspectives on 1) malaria vector control interventions, progress, and challenges; and 2) alternative uses and disposal challenges of the old LLINs 3) WHO guidelines on awareness of proper management of LLINs. The interviews were also conducted in Swahili and audio-recorded; alongside with this, specific notes were taken.

### Data processing and analysis

All survey data were extracted from Kobo ToolBox software [30], checked and cleaned in Excel, and finally coded in R statistical software version 4.1.2 [31]. The data analysis was mainly descriptive statistics, and variables were summarized using frequencies and percentages.

Audio data from FGDs and KIIs was transcribed and then translated from Swahili to English. Notes taken during the discussions were incorporated into the written transcripts. The transcripts were then imported into Atlas.ti.22 software [32] for coding. Survey findings informed the development of KII and FGD guides, and then the guides informed deductive coding.

Findings were presented using the integration principles and practices in mixed-methods designs as described by Fetters *et al*., [33]. A weaving approach was used, in which both qualitative and quantitative findings were reported together based on the illustrated themes in Fig 2. Quantitative findings from the survey were presented, and explanations for some of the concepts were given from the FGDs. Direct quotations from the FGD participants were reported in some selected cases to further describe the themes.

### Ethical considerations

Ethical approvals to conduct this study were provided by the Medical Research Coordinating Committee of the National Institute for Medical Research of Tanzania (NIMR) with approval number (Ref: NIMR/HQ/R.8a/Vol. IX/3353) and the Institution Review Board (IRB) of Ifakara Health Institute with approval number IHI/IRB/No:12–2022. Before conducting the study, the district medical officers were informed about the study, and they granted permission and informed all local leaders through introduction letters Ref No. IHI/ADM/22/0689 and IHI/ADM/22/0690. Additionally, consent to conduct the study was sought from both communal and individual levels. Community consent was obtained from face-to-face discussions with local leaders about the study and requests to conduct it in their wards. Individual consent was obtained by discussing with each participant about the study procedures and their implications and importance, followed by a request to participate. Those who agreed to participate were given written consent forms to fill out before filling in the questionnaires. Permission to publish this manuscript was obtained from the National Institute of Medical Research (Ref: NIMR/HQ/P.12 VOL XXXV/77).

## Results

### Socio-demographic characteristics of the study participants

A total of 384 household representatives (192 from peri-urban areas and 192 from rural areas) responded to the survey questionnaire, of which 67.5% were female and 32.5% were male. Regarding occupation status, 359 were farmers, less than 12% engaged in business activities, and fewer (8.0%) engaged in other activities. More than three-quarters of the participants (83.3%) had a primary education; 16.7% had a secondary education and above. Nearly two-thirds (67.7%) of the participants were married or living with a partner, 23.3% were unmarried, and 9.1% were widowed. More than 50% of the interviewed households had a household size of 4 to 6 people (Table 1).

**Table 1:** Demographic information of the study participants (N = 384).

Variable	% (n)
Gender	
Male	32.5 (125)
Female	67.5 (259)
Age group	
18–40 Years	49.2 (189)
41–60 Years	34.4 (132)
Above 60 Years	16.4 (63)
Location	
Peri-Urban	50.0 (192)
Rural	50.0 (192)
Marital status	
Married/Cohabited	67.7 (260)
Unmarried	23.2 (89)
Widowed	9.1 (35)
Educational status	
Primary	83.3 (320)
Secondary and above	16.7 (64)
Main occupation	
Employed	2.9 (11)
Farmer	93.5 (359)
Fisher	1.3 (5)
Unemployed	2.3 (9)
Household size	
1–3 people	36.4 (140)
4–6 people	50.3 (193)
Above 6 people	13.3 (51)

Values are reported as %(n)

### Primary themes generated

The analysis of the FGDs and KIIs scripts revealed the presence of four key themes: 1) general knowledge on malaria prevention measures; 2) what happens past the useful phase; 3) awareness and perceptions of WHO guidelines; and 4) recommendations for improving disposal mechanisms of LLINs in the future.

### General knowledge on malaria prevention measures

Almost two-thirds of the respondents, 62.2% (n = 384) had bed nets, particularly long-lasting insecticidal nets (LLINs), and used them on a regular basis. While 37.8% (n = 384) did not possess LLINs, this group includes individuals with untreated nets (non-LLINs) and those without any bed or mosquito nets (Table 2). The respondents were aware of malaria transmission and appropriate protective measures due to extensive awareness campaigns by the Tanzanian Ministry of Health and non-governmental organizations. The most commonly reported prevention measures are LLINs, mosquito coils, skin mosquito repellents, long clothes, larval source management (LSM), and aerosol sprays. Other preventive tools mentioned were smoky fires from specific leaves or barks and burning of unused clothes to chase away mosquitoes for some time before bedtime. The community members mostly used bed nets indoors with minimal protection outdoors, as this community member said:

**Table 2:** Malaria prevention methods, nets changing habit, existing LLINs alternative use practices and their causes.

Variable assessed	Participant responses	% (n)
LLINs ownership (n = 384)	Yes	62.2 (239)
	Untreated nets and no nets at all	37.8 (145)
Number of LLINs owned (n = 384)	<2	54.2 (208)
	≥2	45.8 (176)
Bed nets changing frequency (n = 239)	Once 6–12 months	34.3 (82)
	Once 1–2 years	38.1 (91)
	Once >2 years	13.0 (31)
	Forgot	2.5 (6)
	Never changed	12.1 (29)
Bed nets changing decision (n = 239)	LLINs being worn-out	43.5 (104)
	LLINs being torn	50.2 (120)
	Other reasons	6.3 (15)
Existing alternative uses of LLINs (n = 239)	Ropes for tying and covering items	21.0 (50)
	Crops and seedlings fencing	7.5 (18)
	Chicken coop	5.0 (12)
	Other alternative uses	7.5 (18)
	No alternative uses	59.0 (141)
Causes of alternative uses (n = 239)	LLIN being worn-out	12.6 (30)
	LLIN being torn	23.0 (55)
	Other reasons	5.4 (13)
	No alternative uses	59.0 (141)

Values are reported as % (n)

*“First of all*, *I always ensure that the bed net is tucked in by 06*:*00 PM in the evening to prevent mosquitoes from entering into the bed net*. *While we are outside*, *we do not use any specific method to protect ourselves*, *we only wave to chase away mosquitoes*, *even though they continue to bite us*. *Once we are done with the outdoor activities*, *we go inside and sleep under LLIN*, *trusting that there are no mosquitoes” (Male*, *Kivukoni)*.

### Perceptions of community members on LLINs uses, effectiveness and challenges

Many participants mentioned acquiring their bed nets through purchases (31.5% n = 384), while others acknowledged receiving them as donations or aid (30.7%; n = 384) from clinics or school programs targeting primary school children. Despite the availability of these donated LLINs, most participants expressed a preference for using the bed nets they had purchased, although most of them were untreated. The primary factors deterring using aid-provided LLINs include their smaller size relative to their beds, a tendency to tear quickly, and the tendency to shrink after stitching when damaged.

Bed nets have been reported as effective malaria control method. The majority of FGD participants had a positive attitude towards bed net use, although they expressed frustration that malaria remained a problem despite the regular use of LLINs. The participants’ responses to the effectiveness of LLINs in malaria prevention were more salient subjective factors. They claimed that the effectiveness of LLINs was inhibited or reduced in several circumstances, including sitting outside during the evening and the need to run chores, which may result in outdoor mosquito bites, as this community member reported:

*“In my understanding*, *the use of LLIN can be effective*, *but it depends on the circumstances*. *For example*, *staying outdoors in the evening while doing chores exposes one to being bitten by mosquitoes before entering the bed net” (Female*, *Katindiuka)*.

Furthermore, some participants argued that depending on the LLIN alone is not a solution to prevent one from getting malaria, especially if the LLIN is not well cared for or repaired, mosquito breeding sites are not eradicated, and doors and windows are left open until sunset.

*“In my opinion*, *LLIN is not an effective way to prevent malaria because if you only use LLIN while your outdoor environment is not clean and mosquito breeding sites are not cleared*, *even though you sleep under LLIN*, *you do not prevent malaria” (Female*, *Katindiuka)*.

The majority of participants had a positive attitude towards sleeping under LLINs to avoid mosquito bites and malaria infections. However, there were some challenges in using bed nets on a daily basis, particularly LLINs. These include LLIN durability, large mesh (holes), small net size, and an irritating odor when the LLIN is new, triggering a cough-like reaction. Participants expressed dissatisfaction with some LLINs due to unfriendly characteristics, such as their large mesh size, which is perceived to allow small mosquitoes to penetrate through. Furthermore, when repaired, LLINs tend to shrink and shorten, making it difficult to keep securely tucked in sleeping beds or mattresses.

*“The donated LLINs have such large mesh or holes that even the tiniest mosquitoes can get through*. *You might think you have protected yourself from mosquito bites*, *but by the time you realize it*, *the mosquito has already fed on you*. *So*, *you end up wondering how it got inside*. *Before going to sleep in the LLIN*, *I always ensure it’s properly tucked in and chase away all the mosquitoes inside” (Female*, *Minepa)*.

### Bed nets replacement habit

Most of the participants mentioned replacing their bed nets every 6 months to 3 years, primarily based on the presence of tears or damage. Primarily, it is perceived that the effectiveness of LLINs in mosquito protection diminishes within the first 6 months, often due to visible holes or tears, particularly when used by children, as these nets tend to have a shorter lifespan. The participants’ responses to changing their bed nets were similar across all focus groups. The primary factors included the presence of many holes in the LLIN, visible tearing, and the fading of the insecticide, rendering the bed net unsuitable for its intended purpose of protection (Table 2). Although most of the FGD participants stated that the age of the LLIN was not the reason for them to change their nets as long as they were still in good condition (referring to the number of holes), repairs were made on the nets in the absence of new bed nets to replace them for economic reasons.

*“I change them three times a year*. *My criteria for replacement are*: *first*, *when the nets wear out*, *meaning when they have holes in them*, *I replace them with new ones*. *Secondly*, *it depends on the children’s usage*, *as children tend to misuse the nets based on their environment*, *so they get damaged quickly*. *Therefore*, *if I wait and change them only twice a year*, *the children are at risk of getting malaria” (Female Mlabani)*.

Participants also pointed out that another factor influencing the replacement of bed nets was the seasonal variation, particularly between dry and rainy seasons. They noted a significant reduction in mosquito breeding sites during the dry season, whereas mosquito numbers surged during the rainy season. Consequently, the rainy season became the primary motivation for them to replace their bed nets due to the rapid increase in mosquito populations resulting from a higher number of breeding sites compared to the dry season. Additionally, a desire to repurpose the old nets for alternative uses influenced the decision of a few participants to change them.

*“I normally change the LLIN twice a year*. *The reason that I change the LLIN twice is when the rainy season starts since there is an increase in mosquito population compared to the dry season*. *You can remain with the bed net regardless of whether it is torn during the dry season*, *but immediately when the rainy season commences*, *I change the LLIN” (Male*, *Mlabani)*.*” (Male*, *Mlabani)*.

*“I replaced my LLIN because*, *when the fishing season began*, *I found the old net no longer served its purpose*. *Consequently*, *I repurposed it for fishing and purchased a new one for mosquito protection” (Female*, *Katindiuka)*.

### Disposal practices of old LLINs and challenges

More than half of the participants reported disposing of their bed nets when they are old or worn-out 57.8% (n = 384). The most commonly reported disposal mechanisms were burning 30.7% (n = 384), disposing with other garbage 14.8% (n = 384), alternative uses 8.6% (n = 384) and 3.7 others (Table 3). In the focus group discussion, participants confirmed that the entirely worn-out LLIN should be burned to ashes because it was useless; therefore, it should be burned along with other household garbage or waste. The participants explained that they burn the old nets to reduce environmental pollution as they do not disintegrate easily when left out or buried, as one community member said:

**Table 3:** Disposal practices and WHO guideline awareness on the proper disposal of old LLINs.

Variable assessed	Participant responses	% (n)
Existing disposal practices of LLINs (n = 384)	Burning	30.7 (118)
	Garbage pit	14.8 (57)
	Alternative uses	12.2 (47)
	Never disposed of	42.3 (162)
Reasons for improper disposal of LLINs (n = 384)	Limited understanding on proper disposal of LLINs	60.4 (232)
	I don’t have LLINs	39.6 (152)
Perceived side effects of poor disposal of LLINs (n = 384)	Environmental pollution	66.9 (257)
	Harmful to humans and non-humans	10.2 (39)
	I do not know any side effect	22.9 (88)
Prevention of LLINs improper disposal (n = 384)	Yes	82.1 (315)
	No	17.9 (69)
Presence of LLINs collection intervention (n = 384)	Yes	2.9 (11)
	No	97.1 (373)
WHO guideline awareness (n = 384)	Yes	2.6 (10)
	No	97.4 (374)
WHO guideline willingness to comply (n = 384)	Yes	93.2 (358)
	No	6.8 (26)
LLINs collection strategy opinions (n = 384)	Set a designated area for the collection of LLINs	29.2 (112)
	Awareness promotion on proper disposal of LLINs	12.2 (47)
	Compensation for worn-out LLINs	52.6 (202)
	I do not know	6.0 (23)

Values are reported as % (n)

*“It is just like any other waste; you just burn them down; you burn them on the garbage pit*, *or if you do not have a garbage pit*, *you dig it up and burn the LLIN with other waste” (Female*, *Minepa)*.

Some participants reported that they were unaware of the whereabouts of their old bed nets after buying or receiving the new ones. However, one community member mentioned dumping the nets into the water body (river) when they are no longer useful for fishing purposes. This might pose a danger to some aquatic biodiversity, as stipulated by SDGs and environmental authorities:

*“Furthermore*, *to illustrate*, *aside from utilizing them in gardening*, *individuals who engage in fishing often do not bring these nets back home once they are no longer needed*. *Instead*, *it’s common to find someone submerging them in the river” (Male*, *Mlabani)*.

### Alternative uses of old LLINs

**Fig 3:**
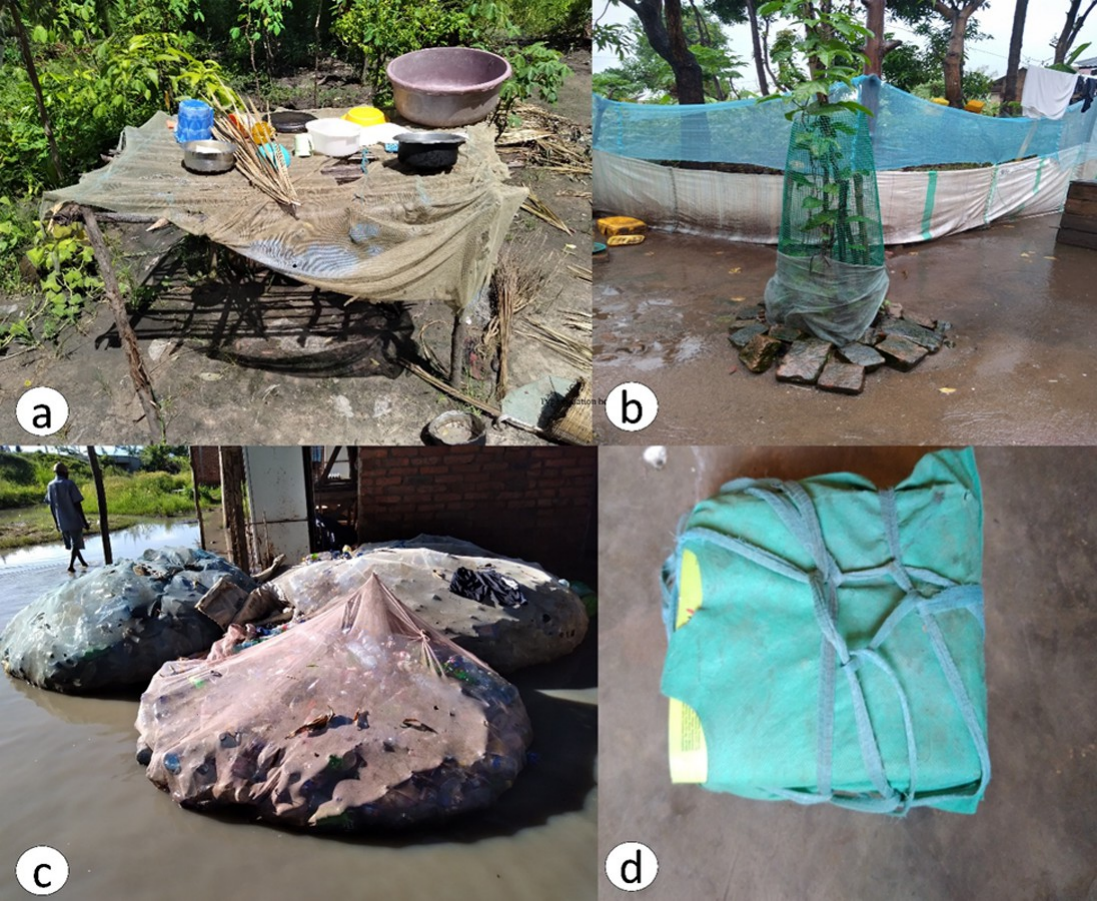
Typical examples of existing alternative use in the study sites, **a**) Illustrates a piece of bed net draped on top of the hut used for drying utensils, **b**) illustrates pieces of bed nets stitched together used for fencing the seedling and garden, **c**) illustrates bed net used as a carrier for the collected recycled plastic bottles and **d**) shows bed net being used as ropes to tie up pupil’s books as one baggage easily to carry.

During the focus group discussions, participants reported that LLINs that are due for disposal are either torn or worn out; therefore, these can be put to alternative use as an indication that the useful life of LLINs has ended. In all focus groups, the length of the bed net’s lifespan was defined by its physical condition to provide mosquito protection and its socioeconomic values based on alternative uses. From the survey, respondents reported several LLINs whose alternative uses had previously been seen or practiced in their villages, including crops and seedling fencing (7.5%; n = 239), chicken coops for protecting chickens from predators (5.02%; n = 239), fishing activities, and ropes for tying items (20.9%; n = 239), as summarized in Table 2 and Fig 3. Other minimal alternative uses (7.5%; n = 239) included curtains, recycled bottle carriers, washing sponges, bathing sponges, floor scrubbers, and dish dryers. The most dominant LLIN alternative use reported across all focus groups was nets being used for making ropes due to their sturdy nature for a variety of needs, such as ropes for building houses or huts, curtains, clotheslines for hanging clothes, and ropes for tying different items. For example, those who sell charcoal sew charcoal bags with LLIN.

*“There is a time when we use LLIN for building our houses or huts because the ropes are a little firm; we tear them apart like ropes*, *and then we use them to tie poles for building houses" (Male*, *Kivukoni)*.

It was further reported that LLINs are used for fencing or as protective barriers for gardens around crops, tree seedlings, and chicken coops. When employed for garden fencing, their purpose is to shield crops and tree seedlings from potential destruction by chickens. It is also used to make scary clowns on rice farms to chase away birds. On the other hand, the chicken coop is used to protect chicks from predators such as hawks.

*“For me*, *when the LLIN is worn out*, *I use it for fencing the chick coop at home*. *Sometime when I grow a garden*, *I use LLIN to fence the garden to prevent chickens from destroying the crops” (Male*, *Kivukoni)*.

Another dominant alternative use is fishing, which is an important livelihood activity for communities near the Kilombero River. LLINs were actually preferred to trap small fish, which in turn are used as baits to catch larger fish. The practice appears to be widespread in Kivukoni village, which is located along the Kilombero River.

*“During rainy seasons like right now*, *we use LLINs for fishing at the river*. *About 80% of the residents of this valley use LLINs for fishing because we are residing along the river valley” (Male*, *Kivukoni)*.

### Improper disposal of LLINs and motives for alternative uses

Major motives for alternative uses of the nets were LLIN being torn 23.0% (n = 239), LLIN being worn-out 12.6% (n = 239) and other reasons 5.4% (n = 239) such as limited understanding of its proper disposal, alternative materials and having more than one net. Although, from the FGDs with community members, numerous reasons emerged regarding motives for alternative uses of LLINs. The major motive was LLIN fabric material characteristics considered to be sturdy and durable to fit for other purposes hence saving costs as one community member said:

*“These LLINs*, *if you touch them*, *are rigid*, *if you look closely*, *they are the ones that we use for alternative uses especially for fishing*, *chicken coop and fencing the garden*, *they are used because of their steadiness” (Male*, *Mlabani)*.

Another dominant motive for the repurposing of the old LLINs for alternative uses was poverty; this was echoed by the majority of participants across all the focus groups. In order to save on costs for working tools such as fishing nets and mesh for fencing to protect chicks and gardens, LLINs were used as alternative materials. For that reason, participants voiced using LLINs as alternative materials for income generation, such as fishing, in order to purchase goods and other basic needs such as paying bills, paying for food, and paying school fees. Due to these scenarios, multiple respondents reported turning to LLINs for alternative uses.

*“Poverty becomes evident when an individual desiring to engage in fishing lacks the means to purchase fishing nets*. *In such circumstances*, *they resort to using the LLIN for fishing in order to generate income” (Female*, *Minepa)*.

Lack of disposal knowledge and the adverse effects that could result from the repurposing or poor disposal of LLINs are indicated as the major perpetuators of the poor disposal and alternative uses of the old LLINs. This was audibly echoed by almost all participants to be the major reason for the ongoing alternative uses and poor disposal of the old LLINs. Lack of awareness and knowledge on what to do with the old LLINs were stated to be the motives for alternative uses and poor disposal of the old LLINs by the majority of the participants.

*“The main reason for all this is a lack of education*. *If these people were sensitized to the proper and improper uses of old nets*, *they would not do that*. *In this world*, *even if education is given*, *the response cannot be 100%; there are people who will always be different and resistant*, *but we will continue to educate each other slowly” (Male*, *Mlabani)*.

Another reported reason that perpetuates the alternative uses is having more than one bed net. It was noted that a single household received LLINs from multiple sources; for example, pregnant women could get LLINs from clinics, children from their school, mass distribution from the government, and ongoing research projects. Some people would tear the LLINs and join them to form the desired size or shape that fit their purposes. Others with multiple bed nets or whose beds were bigger than the received bed nets and had no other activities would sell them to those who needed them, particularly for alternative uses.

*“I have personally observed this problem*. *I witnessed it when the donated bed nets were distributed; some individuals received four*, *while others got five*. *There were men actively seeking bed nets*, *even purchasing them for their fishing needs*. *New bed nets were distributed with the intention of providing mosquito protection*, *but some recipients ended up selling them to those in need of fishing*.*” (Female*, *Katindiuka)*.

### Perceived adverse effects related to improper disposal and alternative uses of LLINs

The majority of the participants were entirely aware of the harm that poor disposal or repurposing of LLINs could cause the environment and living organisms. They expressed fear of the potential health risks and environmental pollution from the remaining insecticides embedded in the LLINs. Among the potential adverse effects captured during the focus group discussion were soil and water pollution, killing of living organisms, air pollution when these LLINs are burned on the open space, and mosquitoes developing resistance to the insecticide. The majority of participants expressed their deep fear and concern about the side effects when LLINs are used for fishing activities, saying that they could kill untargeted organisms and premature fish. Consequently, there could be ongoing health risks or disease outbreaks related to the insecticides, which people are not aware of, resulting from consuming fish that are trapped using LLINs.

*“I would like to emphasize the need for a proper system to control the haphazard disposal of bed nets*, *as my colleagues mentioned earlier*. *These bed nets*, *if simply discarded*, *do not decompose; they essentially do not rot*. *When you throw them away*, *they remain intact*, *almost like a layer of tar*, *even with holes in them*. *This can be harmful to plant growth because plants cannot thrive in areas where these nets have been discarded*. *Moreover*, *the nets contain insecticides*, *and these chemicals can persist in the soil or in the crops we consume*. *They can also directly enter bodies of water*, *where they can harm aquatic life*. *Therefore*, *there is every reason to establish a proper method for the disposal of these bed nets” (Male*, *Mlabani)*.

However, there were different responses among focus group participants, especially males from Kivukoni village who believed the LLINs had no adverse effects since they were perceived to be unfit for mosquito protection. However, this concern could have been subjective because the majority of men at Kivukoni are involved in fishing activities as a source of income. The participants (males from Kivukoni) further justified that if the embedded insecticide within LLIN could not kill mosquitoes, it should not pose harm to human beings and fish.

*“As far as I know*, *there are no adverse effects because these LLINs we are told about have insecticides*, *but in their use*, *we do not see if the mosquitoes actually die*. *Therefore*, *we consider these LLINs to be normal in our uses (alternative uses)*, *and they are safe*. *I believe these bed nets*, *which they say have insecticides*, *are not effective because they are not harmful to the fish*. *We fish using LLINs and eat those fish; these LLINs themselves do not kill mosquitoes; how will they kill me*?*” (Male*, *Kivukoni)*.

### Community awareness, knowledge and compliance to WHO guidelines on proper disposal of LLINs

Among the 384 respondents who participated in the survey questionnaire, only 2.6% (n = 384) reported that they were aware of the WHO guidelines for the proper disposal of LLINs. They primarily learned about these guidelines through radio broadcasts, television programs, and seminars. However, during interviews with key informants and in nearly all the FGDs, it was revealed that neither the key informants nor the FGD participants were familiar with the WHO recommendations for the appropriate management of old LLINs. Furthermore, the FGD participants had never received any instructions regarding the handling of old LLINs, whether when purchasing them from shops or receiving them from clinics or schools. Surprisingly, the majority of participants in the FGDs expressed a willingness to adhere to the WHO guidelines; (93.23%; n = 384) when the facilitator introduced them. Study participants showed a strong willingness to comply with these guidelines and acknowledged their significance in safeguarding the environment, public health, and the well-being of other living organisms, as reported by the community member:

*"I am saying that the recommendations from the WHO are good; firstly*, *they help safeguard our health so that we don’t unknowingly or knowingly fall into these health hazards*. *Another way to collect these worn-out nets is by suggesting that the WHO could work with those distributing these traditional medicines*, *as they often interact with communities every few months*. *If they could use them to collect the old ones from households where they are no longer needed*, *then take them to be safely incinerated at the hospital*, *it could also help in removing them from homes rather than us trying to burn them ourselves" (Female*, *Minepa)*.

Both KIIs and FGD participants appreciated the usefulness of the knowledge regarding the WHO recommendations on the proper disposal of the old LLINs. However, the participants argued and emphasized the use of the local government (ward or village officers) to disseminate knowledge to community members during their regular meetings. This would reinforce the WHO and National Environmental Management Council (NEMC) efforts on environmental conservation to protect public health and biodiversity at large.

*“These recommendations are good and would be useful as instructed*, *maybe we should cooperate with the national malaria control personnel to know the best way to dispose of through burning in high temperature heat in incinerators” (Environmental Officer*, *Ifakara)*.

Nevertheless, a few participants expressed reservations regarding the feasibility of implementing the WHO guidelines, particularly in light of their specific circumstances and practical considerations. They raised various challenges that could potentially impede the successful execution of the collection exercise. Some of the challenges mentioned encompassed a shortage of adequate facilities like incinerators and a lack of strategies for effectively locating and collecting old LLINs at the household level, as a community member said:

*“There is a little bit of a challenge in the recommendations on how to burn those LLINs*. *I see that they have said that they should be burned in high-temperature heat*. *But now if you look for the places that we should use to burn those LLINs*, *it is difficult; the burning facilities of those LLINs are inadequate*, *for example for*, *all Ifakara residents; maybe we all burn them at St*. *Francis Hospital (referring to a local referral hospital at Ifakara town council)” (Male*, *Mlabani)*.

Additionally, the participants emphasized the frequent distribution of LLINs to all community members to ensure accessibility and coverage of LLINs. The participants further argued and proposed the Give and Take (*Nipe Nikupe*) strategy, meaning the national malaria control program (NMCP) team should give new LLINs to the community members in exchange for the old ones, as WHO guidelines suggest to ensure LLIN coverage sustainability but also the removal of old LLINs in the community.

*“When you receive something from someone and leave something in return*, *it’s not seen as taking but rather as an exchange*. *Consequently*, *many people will be more open to the idea*, *especially when they are weary of the old item” (Male*, *Kivukoni)*.

### Recommendation for future: LLINs collection strategies

Participants in the focus group discussions expressed a range of perspectives on the appropriate collection and disposal of LLINs. The primary strategy suggested by these participants involved raising awareness about the potential negative consequences of improper LLIN disposal and promoting alternative uses. It was apparent that community members may not have a clear understanding of how to dispose of old or worn-out LLINs in accordance with WHO guidelines.

There were notable differences of opinion on the proposed strategies among men and women since disposing of garbage is more of a female chore. Some women participants argued that they should be given instructions as early as possible at the LLINs delivery stations, such as clinics, since they are responsible for LLINs care and disposal. Women therefore emphasized awareness creation through media outreach activities and formal in-person sessions, especially in congregational places such as clinics, schools, and village meetings, on what to do with the old LLINs. The public health risks and environmental pollution associated with the negative impacts resulting from the use of LLINs for alternative uses and improper disposal should well be elaborated. This could impact awareness-raising on the proper disposal of the LLINs, as the majority of them did not know what to do with the old LLINs.

*“For example*, *the WHO can establish a system because mothers are heavily involved in these matters*. *When you come to clinics or attend meetings*, *there should be a brief agenda discussing how to properly store these bed nets when they become worn out*, *where they should be directed*, *people should be aware*, *and even the recommended team that comes around should provide a seminar*. *If I have a worn-out net*, *I hand it over*, *and the expert knows what to do with it*. *But also*, *the meetings we often have with people are clinic meetings*, *and we frequently encounter mothers*. *So*, *by using this approach*, *education can easily spread” (Female*, *Minepa)*.

Participants in the focus group discussion suggested that the NMCP team could train the ward or village leaders and community agents for door-to-door collection strategies every few weeks or months to collect the old LLINs from households’ levels to be disposed of (incinerated) in a sound manner. Furthermore, community members proposed other collection strategies, such as replacing the old LLINs with the new ones (“*Nipe Nikupe”*) as WHO guidelines suggested. They also suggest being given an incentive to motivate them to collect the LLINs, as they do with the recycled plastic bottles. It was proposed that the formulation of rules and regulations and their enforcement would improve the outcome. They suggested there should be punishments and fines for community members who do not comply with proper management of the old LLINs. Consequently, this would lead to the eradication of old or worn-out LLIN wastes in the environment and alternative use practices.

## Discussion

The findings suggested that the predominant practices for disposing of LLINs involved either burning them or repurposing them. This was primarily influenced by the lack of official guidelines regarding the appropriate disposal of old or deteriorated LLINs. This lack of clear guidance led community members to dispose of their old or worn-out bed nets based on their own convenience. This is one of the major challenges mentioned that contributed to the proper disposal of these nets and their alternative uses. If there were a clear and well-known guideline, it could help reduce this problem. These LLINs are embedded with pyrethroids and pyrroles; they are also made up of polyester and polyethylene materials, which are non-degradable plastics and may persist in the environment [15, 34]. Therefore, there should be consideration for biodiversity protection as well as water bodies, as stipulated in SGD goals 6 on clean water and sanitation for all, 14 on life below water, and 15 on life on land [13]. The widespread alternative uses of LLINs might pose risks to living organisms. For example, this study revealed community members using LLINs for protecting gardens, fishing, and washing and bathing sponges, so if there are residuals when ingested, they pose a health risk to humans. Additionally, the residual insecticides might hinder efforts towards malaria elimination due to mosquitoes resistance to insecticides and environmental pollution [12].

Despite the presence of the WHO guidelines on handling old LLINs [20], participants, including both public and environmental personnel, indicated their lack of awareness regarding these guidelines. Moreover, they noted that the absence of official guidance and insufficient efforts to promote awareness could hinder the dissemination of these guidelines. However, obtaining clear and easily accessible information from international agencies can prove to be a challenge. Such challenges can complicate the efforts of researchers and practitioners seeking authoritative information. This highlights the necessity for enhanced communication and accessibility in disseminating guidance and research findings in the realm of public health, particularly in matters related to the proper disposal of LLINs.

In contrast to previous studies, this study has shed light on LLIN disposal practices, their alternative uses, and the challenges that hinder the proper disposal of the old LLINs. Encouragingly, despite being unaware of the WHO guidelines on proper disposal of old LLINs, community members were willing to comply with such guidelines. Nonetheless, it’s essential to draw a clear distinction between the stated intentions of individuals to follow guidelines and their actual behavior. To address this, educational programs should be implemented, which not only aim to promote the proper utilization of mosquito nets but also actively monitor and evaluate compliance to ensure their effectiveness.

Regarding LLIN collection strategies, they should be agreed upon earlier with community members prior to the net replacement. Evaluation studies conducted in Tanzania and Madagascar on the collection of expired LLINs demonstrated that collecting worn-out or retired LLINs may be appropriate if community collection strategies are agreed upon prior to the replacement of new bed nets based on community preferences [11]. Hence, it is essential to consider the factors that influence LLIN usage and disposal when developing an LLIN collection strategy. The aim is to extend and improve the retrieval of used or retired LLINs. Therefore, community engagement in various intervention programs has shown significant positive impacts, particularly in the realm of public health initiatives. To develop comprehensive public health intervention programs, it is crucial to involve key stakeholders and the community itself. This inclusive approach allows for a better understanding of local practices and existing interventions, ultimately leading to the creation of sustainable, long-term solutions [35–37].

In Tanzania, research on mosquito control programs has demonstrated the potential benefits of involving communities in efforts to control malaria-transmitting mosquitoes, particularly in Dar es Salaam [37]. Furthermore, community-based approaches have been instrumental in predicting the densities and distribution of outdoor-biting mosquitoes and identifying and characterizing swarms in rural southeastern Tanzania [38, 39]. Eventually, collaborative efforts that engage both government authorities and community members in the design and implementation of projects and programs can lead to smoother operations and the development of sustainable, long-lasting solutions to a wide range of challenges. The study findings suggest that addressing these challenges requires community-based interventions. These interventions include education on the proper disposal of used nets and the promotion of alternative income-generating activities to discourage the misuse of nets.

This study further revealed that bed net physical conditions, such as being torn with many holes or worn out, and the availability of a new bed net were the most important factors influencing bed net replacement. These findings are in line with findings from Akello *et al*., in Uganda [40] one of the reasons for replacing the old bed net was the presence of the new bed net.

Several alternative uses were documented in this study. The most common uses across all study sites were: ropes made from LLINs; crop and seedling protection; chicken coops for protecting chicks against predators; and washing and bathing sponges. Ropes were mainly used for tying, covering items, and hanging clothes. Mostly in rural areas, it was observed that small strips made out of polyester netting were used during house building as ropes tied with branches and wood on mud houses (huts). Similarly, LLINs alternative uses had been documented across other areas in sub-Saharan Africa [11, 34, 41–44]. Numerous motives for bed net alternative uses were revealed from this study, including poverty and the need for alternative materials being the most common, as LLIN is considered to be a sturdy fabric material and durable. Another reason was the lack of official guidance on how to dispose of old LLINs after they expire or are worn out. Several studies previously reported on the main motives for LLINs alternative uses to be economical, and availability of alternative materials, and lack of official guidance [7, 8, 42, 44, 45]. Moreover, the findings from this study shed light on the fact that a significant proportion of bed nets repurposed for alternative uses had reached a state of wear and tear beyond repair or had exhibited perceived reductions in the efficacy of the insecticides, rendering them unsuitable for their primary purpose of protection against mosquitoes. One could argue that the alternative uses, excluding fishing, are reasonable. However, it is imperative to emphasize the necessity of establishing guidelines to regulate these alternative applications. Although the current practices might align with local needs, the implementation of formal guidelines is essential to ensuring that these alternative uses are both safe and environmentally responsible. These guidelines should also address any potential health risks associated with the misuse of bed nets.

Unlike other studies examining the reasons for alternative uses [34, 46, 47], only a small number of community members repurposed LLINs that were still in good condition. They argued that the nets were not suitable for mosquito protection due to their large mesh (holes), smaller size than indicated on the package, and perceived poor quality. It is worth noting that a study conducted in the Solomon Islands had previously reported similar findings regarding bed net preference, with LLINs, especially donated ones (Olyset® Nets), being less preferred due to their unfavorable characteristics, including large mesh size, susceptibility to tearing, and sizing discrepancies [42]. Another factor contributing to the alternative use of LLINs was a sociocultural belief, echoed by male participants, suggesting that insecticide-treated nets might be linked to male impotence or infertility. This misconception aligns with findings from other studies that have also highlighted concerns and misunderstandings surrounding the use of LLINs, particularly regarding potential risks and fears of infertility associated with the insecticides embedded in these nets [48, 49]. Mutalemwa *et al*., proposed that, in order to dissipate these concerns and misconceptions, all bed net users and recipients should be informed that, when used correctly, the insecticides embedded in LLINs kill and repel mosquitoes while remaining safe for humans [11]. Consequently, similar studies have highlighted advantageous alternative uses or repurposing, such as utilizing old nets for window and door screening or to cover house eaves to deter mosquitoes and other insects from entering [7–11]. However, it is important to note that such practices were not frequently reported in the study areas.

The results indicate that the main methods of disposing of LLINs were burning or repurposing, largely driven by the absence of official guidance on how to properly dispose of old or worn-out LLINs. This lack of clear guidance led community members to dispose of their old or worn-out bed nets based on their own convenience. This is one of the major challenges that contribute to the haphazard disposal of these nets, with their alternative uses. If there were a clear and well-known guideline, it could help reduce this problem. These LLINs are embedded with pyrethroids and pyrroles, they are also made up of polyester and polyethylene materials which are non-degradable plastics and may persist in the environment [15, 34]. Therefore, there should be consideration for biodiversity protection as well as water bodies, as stipulated in SGD goals 6 on clean water and sanitation for all, 14 on life below water and 15 on life on land [13]. The widespread alternative uses of LLINs might pose risks to the living organisms for example this study revealed community members using LLINs for protecting gardens, fishing and washing/bathing sponges so if there are residuals when ingested, they pose health risk to humans. Additionally, the residual insecticides might hinder efforts towards malaria elimination due to the mosquitoes resistance to insecticides and environmental pollution [12].

Despite the presence of the WHO guidelines on handling old LLINs [20], participants, including public and environmental personnel, reported not being aware of the guidelines. Furthermore, they reported that a lack of official guidance and inadequate awareness promotion could be barriers to guideline propagation. In contrast to previous studies, this study has shed light on LLIN disposal practices, and their alternative uses and the challenges that hinder the proper disposal of the old LLINs. Encouragingly, despite being unaware of the WHO guidelines on proper disposal of old LLINs, community members were willing to comply with such guidelines. Therefore, collection strategies should be agreed upon earlier with community members prior to the net replacement. Evaluation studies conducted in Tanzania and Madagascar on the collection of expired LLINs demonstrated that collecting worn-out or retired LLINs may be appropriate if community collection strategies are agreed upon prior to the replacement of new bed nets based on community preferences [11]. As a result, the above-mentioned factors should be considered when developing a LLIN collection strategy so as to broaden and scale up the collection of worn-out or retired LLINs.

## Limitations of the study

A significant constraint of this study is its focus on a relatively uniform community in southern Tanzania. This may limit the generalizability of the findings to the entire country or other areas with high household LLIN ownership. Additionally, the questionnaire used in the study did not consider the socio-economic status of the participants, a crucial factor known to influence the utilization of old LLINs.

## Conclusion

The major challenge associated with improper disposal of old LLINs is limited awareness regarding sound LLIN management practices. Therefore, it is essential to prioritize community education on the proper disposal of LLINs. Collaboration between relevant authorities and local communities is of utmost importance in the development of comprehensive strategies for the proper disposal of old LLINs, especially considering the increasing annual distribution of these nets. The establishment of designated collection points is recommended, with a crucial emphasis on consensus-driven collection strategies among local communities before the replacement (recycling) process. This approach would involve residents exchanging their old LLINs for new ones, potentially receiving compensation in the form of either monetary incentives or non-monetary rewards. As a result, these critical considerations should be integrated into policy and program initiatives aimed at solidifying LLINs as a pivotal resource for both malaria control and environmental conservation. Lastly, it is crucial to approach these alternative uses with care and consideration. While repurposing bed nets for various purposes can provide valuable solutions, it must be done in a manner that safeguards their primary function in malaria prevention and respects environmental sustainability. Policies and guidelines should be established to ensure that any alternative uses of bed nets do not compromise their effectiveness in controlling vector-borne diseases or have adverse environmental impacts.

## Abbreviations

DSO: Disease Surveillance Officer
EMO: Environmental Management Officer
FGDs: Focus Group Discussions
ITN: Insecticide-treated Bed Nets
KIIs: Key Informant Interviews
LLINs: Long-Lasting Insecticidal Nets
MFP: Malaria Focal Person
NEMC: National Environment Management Council
NMSP: National Malaria Strategic Plan
SNP: School Nets Program
UNEP: United Nations Environment Programme
WHO: World Health Organization
